# Response to “Global trends in antimicrobial use in food-producing animals: 2020 to 2030”

**DOI:** 10.1371/journal.pgph.0003133

**Published:** 2024-12-30

**Authors:** Samantha Ellis, Jo Coombe, Stephen Page, Jay Gomboso, Rachel Iglesias, Mark Schipp

**Affiliations:** 1 Department of Agriculture, Office of the Chief Veterinary Officer, Fisheries and Forestry, Canberra, Australian Capital Territory, Australia; 2 Department of Primary Industries, New South Wales, Australia; 3 Advanced Veterinary Therapeutics, New South Wales, Australia; 4 Department of Agriculture, ABARES, Fisheries and Forestry, Canberra, Australian Capital Territory, Australia; University of Ottawa Faculty of Medicine, CANADA

We write to draw attention to errors and concerns in the paper by Ranya Mulchandani et al., “Global trends in antimicrobial use in food-producing animals: 2020 to 2030”, published earlier in 2023.

We agree that in the absence of universal data collection and reporting, development of reliable and accurate models to estimate global antimicrobial usage (AMU) is necessary. Model outputs can inform policy decisions, communication materials, and mitigation measures for antimicrobial resistance at both the country and the international level. However, we believe the model developed by the authors and used to produce the results described in their paper is neither reliable nor accurate and therefore inappropriate for these purposes.

We have serious concerns about the underlying assumptions on which the model is based, the paucity and suitability of input data and the conclusions drawn. We discuss each of these issues in turn, then demonstrate the inaccuracies of model estimates of antimicrobial usage at the country level. We have focussed on Australia, however are concerned that these errors may not be unique to the Australian context. We do acknowledge that data limitations were outlined by the authors in the paper, however our concern is that valid conclusions could not be made with the limited data available, and this paper could do nothing more than validate the model, rather than use it to call-out the purported 5 highest users of antimicrobials. Furthermore, we emphasise that papers like this disregard antimicrobial stewardship efforts which are independent to usage levels such as education and prescribing guidelines, and are therefore damaging to global efforts to improve antimicrobial usage monitoring and reporting in animals.

Input data used for this model are sparse. Actual AMU data were available from only 42 countries, which is less than 20% of the countries and territories for which results are presented, and accounts for a greater biomass, therefore likely uses more antimicrobials than other countries. Most of these countries are in Europe, and therefore are not statistically independent due to similarities in socioeconomic factors, climate, farming systems, animal disease incidence and regulatory systems, in comparison to countries elsewhere in the world. This limits external validity, which is the essential basis for predicting usage in countries for which input data was not available. Consider that antimicrobial use in 54 African nations has been estimated using data from a single African country (Cameroon) and use throughout Latin America has been estimated using data from only a single country from that region, Chile. Data from 32 European countries contributed to these estimates. A similar problem arises for livestock species-specific estimates, which are based on data from even fewer countries, around half of which are in Europe. Although they acknowledged these limitations in the text, it does not negate the fact this lack of data would have resulted in significant inaccuracies.

A further concern based on data included for Chile is the significant proportion of amphenicols used compared to other classes, as shown in S2 Fig in the paper. In Chile, 75% of antimicrobial use in 2021 was in aquaculture, and for the amphenicols this was over 90% [1]. Aquaculture was stated to be out of scope for the study. We suspect however, based on S2 Fig, that the method for attributing usage to terrestrial versus aquaculture production systems has not adequately excluded aquaculture use from input data from Chile, which has potentially contributed to biased model outputs.

A number of assumptions are not explicitly stated or justified, in particular those concerned with production intensity. There is no definition given for extensive and intensive systems in this paper, though a previous paper describes ‘intensive systems’ as “high input–high output systems that, compared with extensive systems (backyard production), achieve greater economies of scale and efficiency” [2]. Although it is not explicitly stated, it appears based on S3 Fig that this is only applied to pigs and poultry, not to cattle or sheep. An unstated assumption is that production factors, particularly relating to intensive versus extensive production, are similar between countries with input data and those without. However, there are differences in production and husbandry practices between and within countries that are likely to influence antimicrobial usage in different species which justify consideration of extensive and intensive systems for cattle and sheep. For example, the high estimated usage for sheep (at 243.3mg/PCU) suggests that input data are based on figures from countries with an ‘intensive’ production system. Usage intensity in such systems is unlikely to be replicated in countries with primarily pasture-based ‘extensive’ systems for sheep production, such as Australia. Even within the intensive pig or poultry sectors, there are likely to be differences between countries depending on health status, regulation of antimicrobials and success of stewardship programs that make extrapolation difficult.

One clearly stated assumption is that of a 4-fold difference in antimicrobial usage between intensive and extensive systems, although it is unclear what data supports this. Despite the importance of this and other key assumptions, there is no evidence that attempts were made to confirm their validity or investigate their impacts on model outputs through sensitivity analysis, validation against independent data not used to develop the model, or even informal consultation with appropriate experts.

The model uses data from the World Organisation for Animal Health (WOAH) on regional AMU intensity—expressed in milligrams per population corrected units (mg/PCU)—to adjust country AMU estimates such that average regional usage intensity matches that reported by WOAH. This is likely to introduce inaccuracy at the country level since it is clear from reported values that country usage intensity within a region does not follow a normal distribution. The substantial difference between the median and average values, and the fact that the standard deviation exceeds the mean for most regions (Table 11 of the 6^th^ Annual Report on Antimicrobial Agents Intended for use in Animals [3]) indicates right skewing of the distribution and suggests that scaling using the average will bias estimates upwards, perhaps significantly, for most countries.

Conclusions drawn from model outputs are also concerning. For example, confidence intervals surrounding the estimates for global usage indicate considerable uncertainty and there is substantial overlap for the key time points (68,535–193,052 tonnes for 2020 and 75,927–202,661 tonnes for 2030). It is therefore plausible that global usage could stay the same or even decrease, not only increase as suggested by the authors. Confidence intervals are otherwise not provided in the paper, so it is not possible to assess the uncertainty surrounding other estimates and the degree to which this could impact upon reported outcomes and therefore conclusions.

To demonstrate the impacts of the issues described, we compared the model outputs for Australia as shown in the paper to both unpublished sales volumes (as reported to WOAH) and publicly reported dollar value sales for Australia. The model estimated a total usage for Australia of approximately 4,000 tonnes in 2020, however the actual sales volume was only around 600 tonnes. Dollar value sales, which are published annually [4–13], show a decline in sales from 2010 to 2020 by 8%, when adjusted for inflation. This would suggest a reduction in usage compared to the increase predicted by the model.

**Fig 1:**
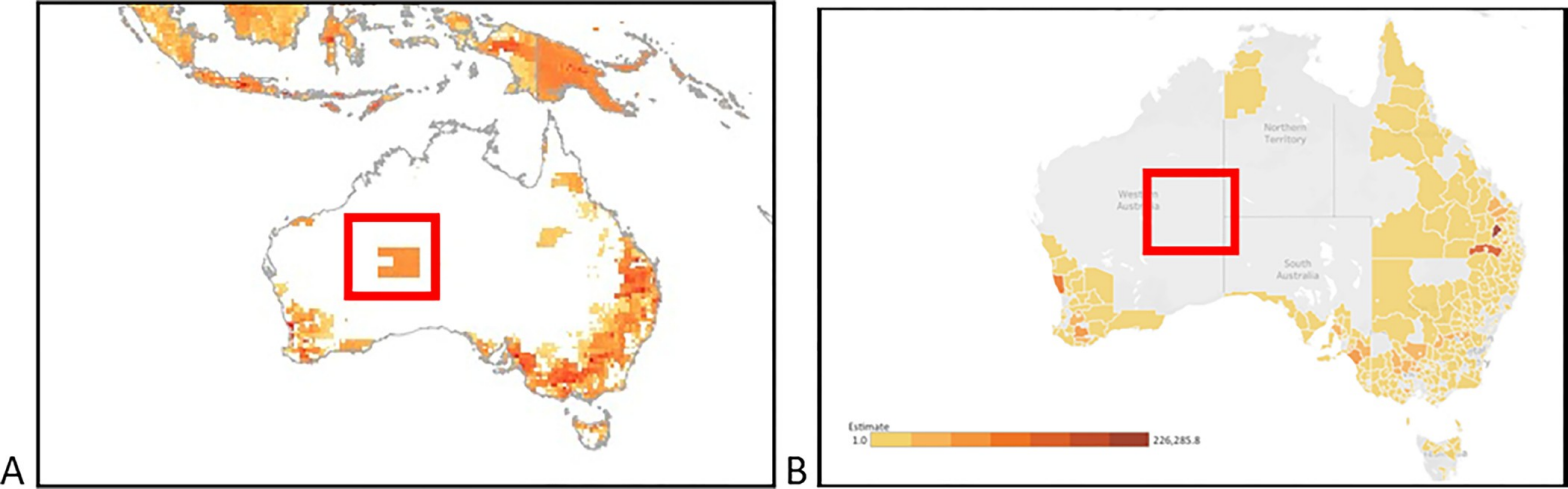
Anomaly in the distribution of domestic pigs in Australia, 2020–2021. Note: Figure shows (a) distribution of antimicrobial usage in domestic pigs as per Mulchandani et al, compared to (b) actual distribution of domestic pig populations in Australia. The red box shows an apparent hotspot in the Gibson Desert Nature Reserve which is not present in actual maps. Source: (A) Mulchandani et al. (2023) [15], (B) ABARES 2023b online dashboard [16].

Model predictions for geographical hotspots of AMU can also be validated against other data sources. For example, in S5 Fig D of the paper, there is an aggregation of AMU intensity for domestic pigs in Australia in a sparsely populated area of desert (Fig 1A). No commercial production for this species exists in this area, as demonstrated by both Australian data sources (Fig 1B) and the referenced input data from Gilbert et al. [14], and it is not clear how the model came to predict a hotspot of usage in this location. Predictions of AMU hotspots for sheep and cattle represent the population distribution of these species in Australia but do not consider production factors that influence the expected distribution of usage intensity. For example, sheep in Australia are raised on pasture and rarely or never in situations such as indoor-housing or lot feeding that are associated with administration of antimicrobials. Similarly, cattle in northern parts of Australia are raised on large pastoral leases where hostile terrain and seasonal conditions such as the wet season may limit stock handling to only 2-3 times per year, making opportunities for administration of antimicrobials very uncommon.

We believe that even our basic attempts to validate the model outputs for Australia demonstrate that estimates are likely to be unreliable for all countries for which there was no input data. The extremely wide confidence intervals for global usage totals and usage trends limit the conclusions that can be drawn.

Furthermore, papers such as this perpetuate a narrative of irresponsibility and inadequacy around animal health sector efforts to contribute to global initiatives against antimicrobial resistance. This narrative fails to take into account the complexity of the policy, regulatory and social factors required for a successful and transparent antimicrobial use monitoring system. It does not recognise the importance of discriminating between appropriate and inappropriate use, nor the steps that have been taken and are continually built upon within the global animal health sector to strengthen antimicrobial use monitoring.

Finally, this paper reinforces and exacerbates stakeholder concerns that any antimicrobial use in agriculture will be misconstrued as excessive or inappropriate, and does not recognise the importance of legitimate use of antimicrobials to safeguard the health and welfare of animals.

Politicians, journalists, and the public have become used to considering the outputs of models as an accurate reflection of reality, as was clearly seen during the recent COVID-19 outbreak. They may not have the knowledge and skills to critically analyse methods and assumptions and will tend to adopt and propagate headline conclusions regardless of accuracy. It is therefore incumbent upon authors to carefully consider their study design, explicitly state and cross-check their assumptions, validate outputs, conduct sensitivity analysis and be conservative in drawing conclusions.

Governments and regulatory authorities rely on credible, robust evidence-based research to inform policy. It is therefore essential that research does not send misleading signals regarding where resources need to be focused.
